# Functional association between telomeres, oxidation and mitochondria

**DOI:** 10.3389/frph.2023.1107215

**Published:** 2023-02-20

**Authors:** Efthalia Moustakli, Athanasios Zikopoulos, Prodromos Sakaloglou, Ioanna Bouba, Nikolaos Sofikitis, Ioannis Georgiou

**Affiliations:** ^1^ Laboratory of Medical Genetics, Faculty of Medicine, School of Health Sciences, University of Ioannina, Ioannina, Greece; ^2^ Obstetrics and Gynecology, Royal Cornwall Hospital, Truro, United Kingdom; ^3^ Department of Urology, Ioannina University School of Medicine, Ioannina, Greece

**Keywords:** telomere, sperm telomere length, mitochondria, reactive oxygen species (ROS), epigenetics (DNA methylation), male infertility, inositol, antioxidants

## Abstract

Prior research has substantiated the vital role of telomeres in human fertility. Telomeres are prerequisites for maintaining the integrity of chromosomes by preventing the loss of genetic material following replication events. Little is known about the association between sperm telomere length and mitochondrial capacity involving its structure and functions. Mitochondria are structurally and functionally distinct organelles that are located on the spermatozoon's midpiece. Mitochondria produce adenosine triphosphate (ATP) through oxidative phosphorylation (OXPHOS), which is necessary for sperm motility and generate reactive oxygen species (ROS). While a moderate concentration of ROS is critical for egg—sperm fusion, and fertilization, excessive ROS generation is primarily related to telomere shortening, sperm DNA fragmentation, and alterations in the methylation pattern leading to male infertility. This review aims to highlight the functional connection between mitochondria biogenesis and telomere length in male infertility, as mitochondrial lesions have a damaging impact on telomere length, leading both to telomere lengthening and reprogramming of mitochondrial biosynthesis. Furthermore, it aims to shed light on how both inositol and antioxidants can positively affect male fertility.

## Introduction

Telomeres are ribonucleocomplexes at the ends of chromosomes that protect the genome from deterioration, undesirable recombination, and altered gene expression. These are recognized as main contributors to genome integrity. Telomere length, in human somatic cells, ranges from 5 to 15 kb, whereas in germ cells it is equal to 10 to 15 kb. They are structures consisting of non-coding “TTAGGG” repeats, which are particularly rich in guanine making them vulnerable to oxidative damage. According to *in vitro* research, oxidative stress accelerates telomere shortening and decreases telomerase activity (1). As telomere length declines with aging, age-related diseases such as cancer, diabetes, and cardiovascular disease have all been linked to it. In general, the functional properties of chromosomal DNA are impaired by the shortening of telomere length. The potential contribution of telomere length to reproductive aging is generating significant interest and stimulates research effects (2). According to telomere-based theory, there should be a biological marker of aging in reproductive aging as telomeres now play a variety of new roles in germ-line cells (3). Studies that have examined the function of sperm telomeres in male fertility and reproduction have found a correlation between sperm telomere length and sperm quality, DNA integrity, and age (4, 5).

Telomere length is known to vary individually and to decrease during telomerase's hiatus following each cell division, which ultimately causes replicative senescence (1). However, rather than the mean telomere length, the percentage of short telomeres is the only component required to predict lifetime (6). The enzyme telomerase has the ability to control the gradual telomere shortening brought up by the end-replication machinery. In order to prevent the addition of new repetitive sequences, telomerase, a holoenzyme, adds the repeated sequences at the 3′ end of telomeres (1). As a holoenzyme that adds repeated sequences to the 3′ end of telomeres, telomerase prevents the insertion of new repetitive sequences while maintaining the telomeres' ability to function. Telomerase is a ribonucleoprotein that includes two subunits; the telomerase reverse transcriptase (TERT) subunit and the telomerase RNA (TERC) subunit.. A single strand of telomeric DNA that is complementary to TERC is generated by the enzyme TERT and attached to the 3′ overhang. The DNA replication machinery synthesizes the trailing strand using the previously formed telomeric DNA strand. When telomerase activity is inactive, telomeres and subtelomeric regions use alternative lengthening of telomeres (ALT) by homologous recombination to preserve their length (7). Recently, it was revealed that TERC does indeed have a telomerase-independent role. Telomere maintenance is not only controlled by nuclear processes, according to recent findings (8). Signals induced in the cytoplasm control nuclear activities, as that is the movement of proteins, metabolites, and molecular signals between the nucleus and mitochondria are only a few well-known examples (9). These co-regulations may be transitory or only apply to certain conditions. Telomere maintenance, subcellular localization of telomerase subunits, and mitochondrial activities are all closely related. According to earlier research on aging, oxidative stress and inflammation caused by mitochondrial malfunction may speed up the loss of telomeres in tissues that have completed the mitotic cycle (10). Therefore, patients who have significant oxidative damage may develop early aging illnesses and cellular senescence (11).

## Sperm telomeres, chromatin and mitochondria

The answer to the question of whether telomere length could serve as a diagnostic tool for evaluating sperm quality and fertility is a contentious one in the study of male germ cells' telomeres. Sperm chromatin quality isn't regarded to date, as a significant factor in male infertility by the World Health Organization (WHO) (12). The suitable sperm parameters are listed in accordance with the lowest reference limit (Table 1). However, a small number of studies have found a link between male infertility and sperm telomere length (STL), indicating that the chromatin quality of sperm should be taken into account (13, 14).

**Table 1:** Antioxidants as a treatment in male infertility and their activity pattern in the sperm cell. The *in vivo* and *in vitro* results are presented in the following table.

Antioxidants	Mechanism of Act	Results In Vivo	Results In Vitro
**Vitamin E**	Neutralises free radicals (Hsieh et al 2002)	Improvement in sperm motility (Souleiman et al 1996)	Improvement in the binding to the zona pellucida of the unfertilized human oocyte in a competitive zona binding assay (Kessopoulou et al 1995)
**Vitamin C**	Neutralises free radical (Akmal et al 2006)	Improvement in sperm motility and morphology when used as an adjunct to varicocelectomy (Cyrus, Kabir, Goodarzi, and Moghimi 2015)	Addition before cryopreservation can reduce DNA damages only in infertile men (Branco, Garcez, Pasqualotto, Erdtman & Salvador 2010)
**Vitamin E** **+** **Vitamin C**		GENERAL OUTCOMES • sperm motility increases 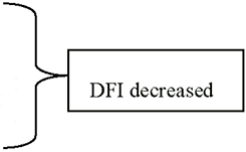 • LPO decreased • Low vitamin C: DFI increased • High Vitamin C: DFI decreased	
**L-carnitine**	Neutralises free radicals and act as an energy source (Showell et al 2014)	Improvement in sperm vitality and motility, increased pregnancy rates (Vicari & Calogero 2001)	Improvement in sperm motility. Patients with lower semen WBC level had significant higher treatment response (Balercia et al 2005)
		• Sperm density and motility increased • DFI decreased	
**Coenzyme Q10**	Scavenges free radical intermediates in mitochondrial transport (Nadjarzadeh et al 2014)	Increased in sperm concentration, motility and normal sperm morphology. Improvement in overall pregnancy rates (Safarinejad, Shafiei 2012)	Improvement in sperm morphology, catalase and superoxide dismutase (Nadjarzadeh et al 2014)
		• Sperm density, motility, TAC increased • ROS level, DFI decreased	
**Vitamin C + Vitamin E + Coenzyme Q10**		• Sperm density and motility increased	
**Folic acid**	Preserves sperm DNA integrity (Wong et al 2002)	Increases in total normal sperm concentration after 26 weeks of treatment in subfertile men (Wong et al 2002)	Low levels associated with increased sperm DNA damage (Box meer et al 2009)
**Carotenoids**	Free radicals scavenging activity (Johnson 2002)	Baseline sperm concentration (no improvement) Higher baseline concentrations had significant improvement with increased pregnancy rates (Gupta and Kumar 2002)	Prevention of decreased motility ischemic/reversion and decreased anomalies in spermatozoa (Hekimoglu et al 2009)
**Selenium**	Aids in sperm motility and OS homeostasis (Ahsan, Kamran & Raza 2014)	Improvement in all semen parameters compared to placebo (Safarinejad and Safarinejad 2009)	Protects sperm plasma membrane from oxidant damage & production of MDA on sperm membrane
**Zinc**	Formation of free oxygen radicals and sperm chromatin stability (Zhao et al 2016)	Improvement in sperm quality, sperm count, progressive motility. Reduction in the incidence of anti-sperm antibodies in male with asthenozoospermia (Omu, Dashti & Al—Othman 1998)	Inhibitory effect of zinc on O2-generation by human spermatozoa and leukocytes (Gavella and Lipovac 1998)
**N-acetyl-cysteine (NAC)**	Free radical scavenging activity (Cifti, Verit, Savas, Yeni & Erel 2009)	Significant improvement in volume, motility and viscosity (Ciftci et al 2009)	Improvement in total sperm motility and decrease in ROS levels (Queda et al 1997)
**Vitamin E + Vitamin C + Zinc + Selenium + L-carnitine + NAC + Coenzyme Q10 + other components**		• Sperm density and motility increased • DFI, ORP decreased	
**Vitamin E + Vitamin C + Zinc + Coenzyme Q10 + L-carnitine + Astaxanthin**		• Total motility and sperm count increased • Sperm density and motility → no change	

It is known that improper DNA packaging can increase the vulnerability of DNA to reactive oxygen species (ROS) that DNA is exposed to, which can cause telomere disruption in mature sperm. Leukocytospermia, a unique cause of male infertility that is directly linked to elevated oxidative stress, is one of the causes (15). Further evidence that there is a relationship between sperm protamination status and telomere length is provided by the fact that sperm with poor DNA quality are susceptible to such oxidative assaults (16). Along with the standard WHO semen characteristics, some investigations have suggested a connection between shorter STLs and infertility or oligospermia. It's interesting to point out that STL and protamination status have a forceful relationship, according to Garolla and colleagues (17). Hence, it has been established that telomere dysregulation in mature sperm is caused by a flaw in chromatin condensation. However, the more disorganized sperm chromatin arrangement can result in higher ROS exposure.

Mitochondria are active organelles located in the cytoplasm of somatic cells, in oocytes, in the midpiece of the spermatozoon, and in other cells and tissues. The quantity of mitochondria in various cell types reflects the need for energy substrate ATP in those cells. The extremely tiny, multicopy mitochondrial genome is exclusively maternally inherited in comparison to the nuclear genome. The 16,569 bp long human mitochondrial genome is a circular double-strand piece of DNA. It contains a non-coding displacement loop (D-loop) and 37 genes coding for two rRNAs, 22 tRNAs and 13 polypeptides (18). Thirteen of these genes code for proteins which are function in five complexes (19). Aside from complex II, all of the mitochondrial complexes have one or more of their genes encoded by the mitochondrial genome. The number of mitochondrial genome copies within the mitochondrion varies depending on the type of cell, with higher ATP users harboring an increased number of mtDNA copies (20).

The regulation of energy metabolism by oxidative phosphorylation plays a key role in mitochondrion activity in cellular function. Energy-dependent cells must have functioning mitochondria because they are the most common energy sources. Instead of turning off the mitochondrial energy metabolism, the most commonly found mutations change the bioenergetics and biosynthetic state of the organelles (21). As a result, it makes sense that they concentrate on the D-loop, because it contains important components involved in the replication and transcription of the mitochondrial genome. Furthermore, mitochondria play a significant part in helping to control a number of physiological elements of reproductive activity, from spermatogenesis to conception. For DNA integrity, sperm motility, hyperactivation, capacitation, acrosome response, and acrosin activity, it is essential that the mitochondrial membrane and the mitochondrial function remain intact (22). Although the range of mitochondrial actions in spermatozoa is yet unknown, conventional mitochondrial activity is necessary for human sperm function and sperm quality. In addition, it has been proposed that the length of the sperm midpiece, represents a battery for the spermatozoon and it is significantly longer in fertile men that in infertile men.

Sperm contains half of the zygote's genetic material and the transfer of sperm DNA should be carried out as intactly as feasible. Sperm telomeres are equally important since, with the exception of the sperm's genome's genes, telomeres appear to be involved in determining the zygote's fate. Sperm telomeres have been closely linked to male infertility during the past ten years and have been highlighted as essential elements for the offspring well being. It was discovered that sperm telomere length ranges from 10 to 20 kb by using validated techniques for measuring sperm telomere length (STL), such as qRT-PCR or quantitive fluorescence *in situ* hybridization (qFISH) (23, 24).

Comparing the telomeres of mature spermatozoa to those of somatic cells, they appear to be longer. In influencing the telomere length in embryos, sperm telomere length (STL) is a significant determinant. Spermatogenesis is a multi-stage process to spermatocytes, spermatids and sperm which involves the proliferation and differentiation of spermatogonia. The earliest phases of spermatogenesis are when telomerase in sperm is activated, and this is also the time when STL is established. They progress to primary spermatocytes and secondary spermatocytes to spherical spermatids before becoming mature, viable sperm. Cellular functioning of mouse purified primary spermatocytes, have significantly larger telomerase activity per cell than cellular function of mouse purified round spermatids. Round spermatids are finally differentiated cells and subsequently they do not undergo divisions and therefore the risk for damage in telomere length is limited (25). Since telomerase stabilizes telomere length, elevated expression of the enzyme has been found during the early stages of spermatogenesis (26). New research suggests that while spermatids and mature spermatozoa have shorter telomeres, spermatocytes retain their full telomere length (27). The final phase is called spermiogenesis, during which the round spermatids undergo a number of striking morphological changes and exceptional chromatin condensation in order to construct mature sperm with a species-specific shape. Specifically, the final phase of spermiogenesis involves numerous chromosomal rearrangements such as protamination (28, 29). Protamination is a process taking place during spermiogenesis, which alters the nucleosome from a histone-based to a protamine-based structure. The repackaging of chromatin maintains 10%–15% of the histones within the cell without voiding the cell completely (30).

In spermatozoa and other spermatogonia that resemble immature germ cells, telomere length is typically the longest. Furthermore, according to numerous studies, older males have telomeres that are longer than younger males. Men who are infertile also have shorter telomeres than fertile controls. As opposed to typical semen characteristics, persons with oligozoospermia actually have shorter sperm telomeres (31). Telomerase activity is significantly increased in men with obstructive azoospermia while telomerase is inactivated during spermiogenesis process. Semen positive for testicular foci of advanced spermatogenesis up to the spermatozoon stage compared to non—obstructed azoospermic males who are negative for testicular foci of advanced spermatogenesis up to the spermatozoon stage (32). In a similar fashion, men with non—mosaic Klinefelter positive for testicular spermatozoa, have significantly higher outcome of testicular sensitive quantity of telomerase assay than males with non—mosaic Klinefelter syndrome negative for spermatozoa (33).

Infertility, unsuccessful fertilization, embryonic lethality, decreased longevity and viability, cell culture arrest, genomic instability, gamete apoptosis, and recurrent miscarriage are all linked to shortened telomere length in germ cells. According to Hemman and colleagues, shorter telomere spermatocytes have a monitoring system that prevents them from completing the final stage of spermiogenesis and instead selectively they undergo apoptosis to eliminate defective spermatozoa or even spermatozoa with shorter telomeres (34). Leukocyte telomere length (LTLs), on the other hand, declines with age, but sperm telomere length increases with increasing paternal age (35). By having an older father during conception, it is inferred that the offspring will have longer telomeres (36). The above support the idea that telomere length is inherited and foresees future generations having longer telomeres. Older men's spermatogenesis appears to have unique mitotic divisions as compared to younger males. As a starting point to explain why older men have longer telomeres, one should consider that the STL variability is influenced by the different ALT mechanisms that each person possesses, as well as potential environmental factors that spermatozoa may have been exposed to (7, 37).

There are two basic theories for telomere lengthening seen in organisms with longer lifespans. First, telomerase has ample time to extend telomeres as the rest of the stem cells deteriorate since it is present in spermatogonia and is active during spermatogenesis up to the elongating spermatids stage. The second hypothesis holds that only germ cells with longer telomeres survive at an advanced paternal age because there is a selection of germ cells based on those with longer telomeres (38). However, as described in round spermatids, mature sperm still have dimeric telomeres, which clash with the former germ cells since telomerase activity has been seen.

**Figure 1:**
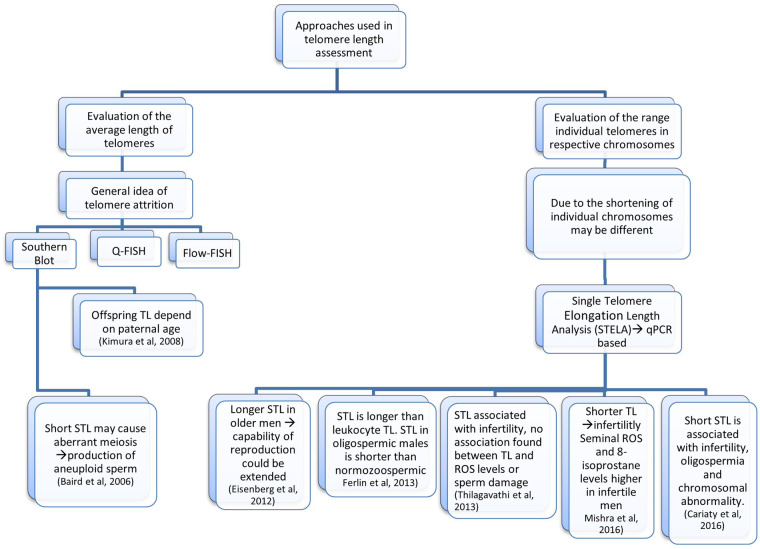
Flowchart of studies that associate telomere length with infertility. In each method the impact on the telomere length is also mentioned.

As more knowledge regarding STLs, male reproduction, and the precise underlying systems has developed, the effects STLs have on reproductive potential have become more apparent. Shorter STLs have been found to be correlated with impaired sperm parameters, such as sperm motility and count (39). Additionally, a correlation between shorter STLs and idiopathic male infertility has been recorded (40) (Figure 1). Long telomeres are typically assumed to be linked to longevity and to act as a protection against the attrition which results from repetitive cell division. Even though the indirect relationship between paternal age and telomere length affects the progeny, it is nevertheless seen as a contentious and divisive topic.

## Effects of ROS on telomeres

The production of reactive oxygen species (ROS), which are primarily produced by leukocytes and immature spermatozoa in the male urogenital tract, is influenced by a number of clinical conditions, including infection or inflammation, varicocele, cryptorchidism, testicular torsion, and toxic exposure (41–44). The overproduction of ROS could result in OS in the seminal fluid. Five types constitute external sources of ROS: lifestyle, environment, infection, iatrogenic, and testicular. The effect of these elements on the generation of seminal ROS has been the subject of numerous investigations. More precisely, ROS are involved in the maturation of post-testicular sperm. The spermatozoa nuclear condensation is strengthened by their participation in the construction of inter-protamine disulfide bridges during epididymal transit (45).

Telomeres are acknowledged to be one of the targets of oxidative stress (46). Given the abundance of telomeres of residues (guanines) at telomeres, which are vulnerable to OS and consequently more likely to cause DNA damage to sperm, infertility is the result (46). Telomere length is known to vary individually and to decrease during telomerase's hiatus following each cell division, which ultimately causes replicative senescence (1). However, rather than the mean telomere length, the percentage of short telomeres is the only component required to predict lifetime (6).

Long telomeres are typically assumed to be linked to longevity and to act as a protection against the attrition which results from repetitive cell division (47, 48). A ground-breaking theory put forth by Moazomian et al. is that mild OS brought on by paternal aging leads to an adaptive extension of telomeres in the male germline, giving growing spermatozoa and the offspring some level of protection from a stressful environment (49). During the fact that telomerase is still active in these cells this is feasible in developing male germline (50). The loss of telomerase activity at this point in development prevents mature spermatozoa from mounting an adaptive, telomere—lengthening response to OS.

Smoking, drinking, exposure to toxins and obesity are just a few of the factors that affect male infertility (51). Numerous studies have found a relationship between the aforementioned parameters and STL integrity (52). These lifestyle factors considerably increase the occurrence of ROS when paired with advanced age (53, 54). Due to their repeated patterns and high guanine content, telomeres are particularly vulnerable to oxidative damage, which results in the formation of 8-oxo-2-deoxyguanosine (8-oxo-dG) (55). Research conducted *in vitro* indicates that oxidative insults are the likely cause of telomere integrity breakdown and telomere shortening (56). Histones may be particularly susceptible to oxidative impact when they are maintained in telomeric loci since they have a low disulfide bond content (55).

The connection between oxidative stress and the length of the telomere in sperm has only been partially studied. It is widely acknowledged that oxidative stress is a major contributor to telomere shortening in somatic cells. By inhibiting the non-random arrangement of mammalian chromosomes in the sperm nucleus, oxidative stress has an impact on the packaging of the chromatin in sperm (57). Increased telomerase signals in the nucleus are caused by a deficiency in chromatin condensation (15). Evolutionarily conserved pathways influence the aging process in certain ways with at least nine having been proposed to date. Genomic instability, telomere attrition, and epigenetic changes are the most prominent signs of aging and have some degree of overlap or connection (58). That is appears logical that evaluating telomerase has a role for the prediction of the presence of testicular spermatozoa in azoospermic men with varicocele (i.e., known to induce intense oxidative stress).

Mitochondrial malfunction is another sign of aging, and as cells have both a nuclear and a mitochondrial genome, this is also intimately tied to genomic instability. The organelles that produce the majority of the intracellular ROS are mitochondria (59). According to the free radical theory, ROS cause DNA oxidation damage in both mitochondrial and nuclear tissues, which results in an accumulation of mutations that eventually lead to aging (60). Nevertheless, it is generally known that this idea is oversimplified because ROS can trigger compensatory pathways that could undo their harmful effects. Since the amount of mitochondrial DNA is a specific indicator of the number of mitochondria per cell, the respective concentration is indicative of cellular bioenergetic capacity also linked to infertility. Apart from the mitochondrial quantity; the integrity is also crucial for infertility (61).

## Seminal oxidative stress

Seminal OS is hypothesized to be one of numerous domain consequences that contribute to the etiology of sperm dysfunction and sperm DNA damage in male infertility (62). ROS formation and ROS elimination by sperm antioxidants are out of balance, which is the main cause of seminal OS. In reality, semen OS levels are not high in fertile men, although they are present in a fourth of infertile men (63). The regulated production of these ROS is necessary for both natural fertilization and sperm physiology including sperm hyperactivation, capacitation, and acrosome response.

Seminal plasma and the spermatozoa themselves are abundant in antioxidants that protect spermatozoa against OS, particularly at the post-testicular stage. Glutathione peroxidase, superoxide dismutase, and catalase are all high-molecular-level enzyme antioxidants found in seminal plasma (64). Men who lack these enzymes are unable to conceive, which damages the DNA in their sperm. The majority of the sperm's antioxidant potential is composed of non-enzymatic antioxidants such ascorbic acid, a-tocopherol, pyruvate, glutathione, L-carnitine, taurine, and hypotaurine which are also found in seminal fluid. Beta carotene, albumin, pyruvate, and ubiquinol are also present in the seminal plasma (65).

Numerous studies have shown that the seminal antioxidant capacity is reduced in infertile men with higher ROS concentrations compared to individuals with normal ROS levels. However, it is still unclear how diminished seminal antioxidant capacity affects sperm failure. It is debatable whether the high ROS concentrations in infertile men's semen are the result of elevated ROS production, decreased ROS removal capacity, or both. In the event that the body is capable of scavenging ROS, dietary antioxidant supplementation may be useful for those likely having the tendency for generating ROS as the smokers do.

Any breakdown in the homeostatic balance between ROS formation and antioxidant capacity in the seminal plasma of human semen leads to OS when highly reactive ROS overwhelms the antioxidant defense systems (66). Sperm DNA fragmentation (SDF) in the nuclei and mitochondria, lipid peroxidation, and apoptosis including mitophagy and lipophagy may all be negative outcomes of high ROS concentration (67).

Although systemic antioxidant insufficiency has not yet been linked to male infertility, it is possible that some infertile men will experience vitamin C deficiency. Researchers have examined the impact of dietary antioxidant consumption (vitamin C, E, and -carotene) on sperm DNA damage so far, but they were unable to find a connection between these variables.

## Mitochondria and human sperm

Human sperm and semen quality depend on optimal mitochondrial activity. For sperm motility, hyper-activation, capacitation, acrosin activity, acrosome response, fusion with the oocyte and DNA integrity, mitochondrial functioning and unharmed mitochondrial membrane potential are substantial. The maintenance of energy generation, necessary for sperm motility is significantly impaired by defects in sperm mitochondrial activity, which may also be the underlying cause of asthenozoospermia (68). The number of mtDNA copies is higher and the integrity of the mtDNA is diminished in males with aberrant semen parameters.

Since human sperm is an extremely specialized and distinctive cell, it has a distinct metabolism that is controlled by certain enzyme isoforms. There are different subcellular sites where glycolysis and mitochondrial respiration take place. Glycolytic enzymes are present on the flagellum, whereas mitochondria are positioned in the sperm's midpiece. Which of the two is the principal source of ATP in human sperm is still debatable (69). According to research on somatic cells, mitochondrial function is compromised by telomere disruption. Human leukocytes from young and middle—aged individuals show a positive association between telomere length and mtDNA copy number. Consequently, age—related changes in paternal variables have an impact on embryo quality.

Sperm hyperactivity and enhanced motility are a direct outcome of sperm capacitation, which increases the conversion of energy substrates into mechanical energy. Spermatozoa must be hyper-activated in order to move through the oviductal mucus and endometrium. When the acrosome reacts, it is also essential to penetrate the oocyte zona pellucida (70). As pointed out, mitochondria convert between 0.2% and 2% of the oxygen taken in into ROS, making them a well-known source of ROS. In low concentrations, ROS are believed to have physiological significance and to control a number of signaling pathways (71). It has been suggested that oxidative stress-induced damage is a common cause of male infertility, and in particular excessive ROS production has been related to this condition. For instance, increased ROS production may result in DNA breakage or lipid peroxidation, which weakens the membrane and lessen mitochondrial activity. Antioxidants have even been suggested as adjuvants for sperm culture medium because of their significant benefits for preserving spermatozoa viability and motility (72).

Globally, sperm abnormalities and problems with sperm motility cause 50% of the infertility cases that affect up to 15% of couples. In over 60% of causes with idiopathic male infertility, sperm motility is a significant contributing factor (73). Due to the phosphorylation of mitochondrial proteins, including signaling proteins connected to chaperons, spermatozoa shape, and sperm metabolism, mitochondria play a major role in controlling sperm motility. Sperm motility is also largely regulated by mitochondria, which are also crucial for capacitation, acrosome response, and fertility. An important structural change that improves the interface between the sperm and the zona is initiated by the phosphorylation of the sperm-surface chaperone. As a result, knowledge of mitochondrial function and activity continues to be one of the key issues in understanding the causes of male infertility (74, 75).

Recent studies have identified a germline aging mechanism that occurs in males is a biological aging process, unique to germ cells (telomere lengthening, higher SDF, extensive DNA methylation alterations) that is linked to older fathers having poor reproductive outcomes. It has been suggested that male infertility or, at the very least, abnormal sperm parameters, may be related to poor overall health. Longer telomeres may be harmful to fertility and may be present in the offspring of aging fathers, although it is unknown how this may affect the overall health of these children (76, 77).

Nonetheless, the growing prevalence of sperm DNA Fragmentation Index (DFI) in older men, which affects approximately 80% of the males in the oldest group, indicates a steady rise in genomic instability in the germline. One of the hallmarks of aging—oxidative stress—has been shown to rise in sperm with age. This may be a factor in the mechanism through which DNA fragmentation increases in older men. Independent of the age of the female partner, the high amounts of DNA fragmentation found in older men's sperm may be responsible for the previously documented longer time to conception and greater loss rates (78).

Most organisms experience a function—dependent decline as they age, which is widely accepted definition of aging. Aging is often caused by accumulation of cellular lesions over time and results in an increase in senescent cells. While cellular senescence and aging are two distinct processes, senescence seems to occur throughout the lifespan which also includes embryogenesis whereas aging is a steady decline over time. Aging is indicated by mitochondrial malfunction. Mitophagy indentifies and eliminates dysfunctional mitochondria. The aging process may be accelerated by increased mitochondrial damage brought on by decreased biogenesis and clearance.

## Mitophagy and telomeres

Apoptosis, which is regulated by numerous cell death signaling and regulatory pathways, is the biologically well-programmed cell death caused by DNA fragmentation. Apoptosis can happen as a result of double strand DNA breakage brought on by ROS. Additionally, ROS causes mitochondrial membrane disruption to generate the signaling molecule cytochrome C that could trigger apoptotic caspases and cause annexin-V to attach to phosphatidyl-serine. Elevated production of ROS in seminal fluid may indicate that infertile patients have considerable mitochondrial damage from high ROS levels (67). In order to maintain cellular homeostasis, autophagy relies on lysosomal degradation, which is a well—conserved catabolic pathway. Both bulk autophagy, which is non-selective, and selective autophagy are possible. Selective autophagy is further subdivided into three groups based on the many substrates that are available: mitophagy (mitochondria), lipophagy (liposomes), and ER-phagy (endoplasmic reticulum) (79). Since mitophagy affects mitochondria and appears to be connected to telomeres, it is the sole topic we specifically address in this review.

First of all, mitophagy contributes to the elimination of the paternal mitochondrial DNA after fertilization and operates through sperm differentiation before fertilization. Endonuclease G has been shown to remove the bulk of sperm mtDNA during spermatogenesis; as a result, the residual vacuolated spermatozoa take part in the creation of the mid-piece of spermatozoa (80). When potentially harmful conditions exist, including oxidative stress, hypoxia, loss of mitochondrial transmembrane potential, anemia, and the accumulation of unfolded proteins, mitophagy actually occurs. Additionally, the energy generated by sperm mitochondria is crucial for both sperm motility and fertilization (81). It is worth noting that the proper deletion of mitochondrial DNA after fertilization benefits both the health of the individual and the homogeneity of the embryo.

Cryptorchidism and asthenozoospermia are intimately related to mitophagy. An increase in body temperature has been linked to both the delay of cryptorchidism spermatogenesis and an increase in the damage to mitochondria that triggers mitophagy, according to recent literature (82). Additionally, the main organelle in sperm that produces ROS is the mitochondria. Apoptosis of the male gamete can be caused by excessive ROS production, which might start mitophagy. Future clinical relevance for the identification and management of male infertility will result from a thorough investigation, with a focus on the relationship between mitophagy and conditions associated with spermatogenesis. The precise mechanism of specific autophagy in spermatogenesis has yet to be demonstrated. The effects of additional types of selective autophagy on male fertility and spermatogenesis must thus be evaluated. Understanding the changes in mitophagy that occur as we age can point to worthwhile research projects to delay aging and improve age-related health consequences. Mitophagy reduces the production of ROS, prevents the accumulation of mtDNA mutations, boosts the production of ATP, and inhibits apoptotic signaling and the activation of inflammatory vesicles.

Telomere biology and autophagy are related, according to earlier research (83–85). Increased cytoplasmic vacuoles and autophagy-related proteins (ATG5-ATG12, LC3-II) are found in cells going through a crisis, which is linked to deprotected telomeres (83). Autophagosomes can be induced and mTOR signaling can be inhibited in malignant glioma cells when telomeric 3′ overhang-specific DNA oligonucleotides, which mimic telomere loop breakage, are used (85). Additionally, TERT binds to and inhibits mTORC1 kinase in numerous cell lines, including HEK 293 T, HepG2, and U-2 OS, leading to the induction of autophagy. Under baseline and amino acid famine circumstances, TERT knockdown causes components of mTORC1 to rise (84). This impairs autophagy. Telomere shortening may impact p53, which is inhibited in part by autophagy and p53 can trigger autophagy as a response mechanism (86, 87).

To maintain physiological homeostasis, cells use an organized and controlled process called autophagy to break down the components of their bodies (88). Extranuclear telomerase, even before the detectable beginning of telomere shortening, is known to have a significant role in the disease-associated shift in the mediator of flow-mediated dilation (FMD). This telomerase action is non-canonical and may reverse coronary artery disease-related vascular dysfunction by triggering autophagy (CAD) (84, 89, 90). Murine embryonic fibroblasts provide support for the hypothesis that telomerase and autophagy interact. TERT over expression enhances autophagy markers, as shown by an increase in the conversion of LC38 I to LC38 II, a sign of enhanced autophagosome formation. In turn, lowering TERT activity decreased the transformation of LC3 I into LC3 II. Autophagy was induced by transgenic over expression of TERT in a mTORC1-dependent manner. Similar to this, Cheng et al. revealed the functional implications of this interaction by demonstrating that inhibition of telomerase inhibits autophagic responses to ischemia-reperfusion within the murine kidney as evidenced by a decreased expression of LC3 II and accumulation of p62, results that were alleviated by treatment with mTOR inhibitor (91).

Recent research has shown that mitochondrial metabolic dysfunction precedes telomere DNA degradation. The presence or absence of a telomere structure is the primary distinction between mitochondrial DNA and chromosomes. However, the D-ring, epigenetic control, G-quadruplex, and heterogeneous double-stranded, supercoiled DNA properties that distinguish telomere DNA from mitochondrial DNA are also present in both types of DNA under replication strain [80]. Telomerase's primary function is to counteract telomere shortening and preserve telomere length. Telomeres also help cells survive by maintaining their length. According to research, telomerase is responsible for removing the oxidative stress-induced subunit TERT from the nuclei and relocating it to the mitochondria. By lowering mitochondrial ROS generation, this co-localization of TERT and mitochondria preserves mitochondrial function and lessens nuclear DNA damage and apoptosis (92, 93). Unexpectedly, cells that totally eliminate telomerase show little or no DNA damage. In contrast, cells with residual telomerase function have an increase in DNA damage. In the event of increasing DNA damage, the authors speculate that telomerase may negatively impact repair enzymes in the nuclei of cells (94). This also explains why, to a certain extent, DNA damage prevents telomerase from continuing to function. In another research study, it has been demonstrated that human fibroblasts expressing TERT can lessen the damage caused by oxidative stress to mitochondrial DNA, which is mostly regulated by base excision repair (BER), and the presence of hTERT does not impair BER repair (95).

Parallel investigations were making the case that aging was not actually brought on by changes to the ends of chromosomes or any other nuclear process, despite what we now know about telomere biology. Instead, these investigations suggested that the primary cause of aging is a reduction in mitochondrial activity or an increase in ROS. A recent study suggests that such association between telomeres and mitochondria may have been present but not yet fully recognized. According to this recent study, telomeres control overall metabolic performance by acting through p53 (96). Making such a connection appears to have brought the mitochondria and the nucleus closer together and help elucidating the fundamental reasons of aging.

## Telomeres and lifestyle

A number of lifestyle choices, including smoking, being overweight, not exercising, and eating poorly, might speed up the shortening of telomeres, putting one at risk for disease and/or dying sooner. Numerous age-related health issues, such as coronary heart disease (97–99), heart failure (100), diabetes (101), increased cancer risk (102, 103), and osteoporosis (104), might manifest themselves earlier in life when telomere shortening is accelerated. Elderly people with shorter telomeres have a much greater mortality risk than those with longer telomeres, according to an analysis of telomere length (105). Multiple aspects of health and lifespan can be impacted by excessive or fast telomere shortening. To the contrary, immortal/cancer cells undergo senescence and/or death when telomere maintenance mechanisms are inhibited and telomere lengthening continues (6).

Telomeres and aging appear to be negatively impacted by smoking and obesity. It has been demonstrated that telomere length and cigarette smoking dosage are inversely correlated (106). In the blood cells of tobacco users, a dose-dependent increase in telomere shortening has been noted (103, 107). According to a study done on white blood cells from females, each pack of cigarettes smoked daily results in an additional loss of 5 base pairs of telomeric DNA, bringing the annual average loss to between 25.7 and 27.7 base pairs (108). The loss of 7.4 years of life due to telomere attrition is the result of smoking one pack of cigarettes each day for 40 years (108). Telomere length has been suggested as a biomarker for assessing the oxidative harm brought on by smoking by Babizhayev et al. (109) and as a potential indicator of aging. Additionally, according to the authors' theory (109), antioxidant therapy can stop oxidative damage that results in telomere shortening. In conclusion, smoking accelerates the shortening of telomeres; increases oxidative stress, and may hasten the aging process.

Enhanced oxidative stress and DNA damage are additional effects of obesity. Waist circumference and body mass index (BMI) strongly associated with higher plasma and urinary reactive oxygen species levels, according to research by Furukawa et al. (110). Independent of age, Song et al. research's (106) has demonstrated a substantial correlation between BMI and DNA damage biomarkers. Obesity-related elevated oxidative stress is most likely caused by an unchecked production of adipocytokines. White adipose tissue from obese mice had higher levels of ROS, but not in any other tissues, showing that the oxidizing agents responsible for the oxidative stress found in plasma could come from the fat tissue. Furthermore, obese mice's white adipose tissue had considerably lower transcript levels and antioxidant enzyme activity than control mice, including catalase and dismutase. According to the related research, enhanced NADPH oxidase pathway in accumulated fat and a lack of antioxidant defense contributed to increased oxidative stress in obese mice. Oxidative stress, which may result in DNA damage, may hasten the shortening of telomeres. Telomere length in obese women is shown to be significantly lower than in lean women of the same age (108). Obesity appears to have a more detrimental effect on telomere length than smoking, with an excessive loss of telomeres in obese people estimated to be equivalent to 8.8 years of life. These findings suggest that obesity affects telomeres negatively and may unnecessarily speed up aging.

Health and telomere shortening rates can both be impacted by the environment. Office workers and traffic police officers exposed to traffic pollution had their leukocytes' telomere length measured by Hoxha et al. (111). The concentrations of benzene and toluene indicated exposure to pollution. In comparison to office workers, the study discovered that traffic police officers' telomere length was shorter in each age group. Similarly, compared to control subjects, the lymphocytes of coke oven workers who had been exposed to polycyclic aromatic hydrocarbons showed greater indications of DNA damage and genetic instability and had considerably shorter telomeres (112). Despite not being associated with age or signs of DNA damage in these workers, telomere length reduction was substantially correlated with the duration of their exposure to dangerous substances. Workers in coke ovens are more likely to get lung cancer, and telomere attrition has been linked to an increased risk of cancer (102, 103). The erosion of lymphocyte telomeres is likewise associated with aging (113). The shorter telomere length in the lymphocytes of coke-oven workers was also associated with hypomethylation of the p53 promoter (112), which may result in the synthesis of p53 (114). P53 expression may limit growth or trigger apoptosis (115). Therefore, exposure to genotoxic chemicals, which may result in DNA damage overall or more severely at telomeres, can accelerate aging and raise the risk of cancer.

Telomere, well-being, and lifespan can all be dramatically impacted by what and how much we eat. Cassidy et al. (116) examined the association between a variety of lifestyle factors and the length of leukocyte telomeres in a substantial population of women. Intake of fiber in the diet was positively correlated with telomere length, but intake of polyunsaturated fatty acids, particularly linoleic acid, and waist circumference was adversely correlated. Reduced protein consumption also appears to lengthen life. Rats lived 15% longer when the protein level of their meal was reduced by 40%. Early in life, rats given a protein-restricted diet showed long-lasting suppression of hunger, slower growth, and prolonged lifespan (117, 118). Telomere length in the kidney of these animals was also much longer. The source of protein also appears to be crucial, as rats whose casein was replaced with soy protein lived longer and developed chronic nephropathies later.

In a study by Farzaneh-Far et al. (119), it was discovered that telomere shortening is associated with a diet high in antioxidant omega-3 fatty acids, and is also associated with a diet deficient in these antioxidants. The length of the telomere and blood levels of omega-3 fatty acids in these individuals were tracked by the researchers over a five-year period. They found an inverse correlation, which shows that antioxidants limit the rate of telomere shortening. Similarly, women who had low-antioxidant diets had shorter telomeres and a moderate risk of having breast cancer, whereas those who consumed high-antioxidant diets including vitamin E, vitamin C, and beta-carotene had longer telomeres and a reduced risk of developing breast cancer (120). The oxidative damage by intrinsic and extrinsic DNA-damaging chemicals can inflict on telomeric DNA may be prevented by antioxidants. According to research by Song et al. (106), exercise duration is negatively correlated with telomere and DNA damage biomarkers as well as p16 expression, a biomarker for aging in human cells. Exercise aids in the mobilization of waste products for faster clearance, reducing oxidative stress and protecting DNA and telomeres. Werner et al. (121) have shown in mice that exercise was linked to increased telomerase activity and suppressed many apoptotic proteins, including p53 and p16. In comparison to non-athletes, human leukocytes obtained from athletes consistently displayed higher telomerase activity and less telomere shortening (121). Exercise may slow the progression of aging and age-related disorders since it appears to be linked to decreased oxidative stress and increased production of telomere stabilizing proteins. Smoking, pollution exposure, inactivity, obesity, stress, and a poor diet accelerate telomere shortening and the oxidative burden. Consuming less food, supplementing our diets with antioxidants, fiber, soy protein, and healthy fats, and engaging in physical and mental exercise are all ways to preserve telomeres, lower the risk of cancer, slow down the aging process, and slow down the pace of cancer development. Telomeres would be protected by these in conjunction with a Mediterranean-style diet rich in fruits and whole grains.

## Antioxidant; male infertility and telomerase

ROS is indeed a key factor in 30 to 80 percent of cases of male infertility. Poor reproduction potential has been linked to excessive ROS and OS production. Both ROS and OS have been linked to SDF, decreased embryo growth and fertilization, low rates of implantation, high rates of miscarriage, and pregnancy loss. To sustain normal cell function, several antioxidant components must be present in the right amounts in each cell type of cell, but these requirements vary depending on the cell type. Antioxidants face the danger of putting patients under reductive stress, which could harm their fertility, if they are given to people who are not experiencing an oxidative attack. To give just one illustration out of many, it was discovered that giving antioxidants vitamins, zinc, and selenium to males with high levels of DNA damage in their spermatozoa caused reductive stress, which can be characterized by a dramatic decondensation of the sperm chromatin. This was most likely caused by the reduction of disulfide bridges in the protamine network that stabile the sperm nucleus.

Unless the homeostatic equilibrium between antioxidants and ROS is interrupted, the male reproductive system would be negatively impacted, leading to the emergence of sperm disorders. ROS have been found to have harmful effects on mitochondrial genome, cell membranes, and apoptotic pathways, not just in sperm. The association involving high rates of ROS generation, and SDF is advantageous since the moderate level of SDF results from excessive ROS production (122). By lowering intracellular ROS production, enhancing mitochondrial respiratory activity and function, and preventing ROS—induced cell death inside the mitochondria, human TERT (hTERT) works to protect cells from OS—induced mortality. Nuclear hTERT is directed to the mitochondria under conditions of low stress and ROS generation because of an N-terminal leader sequence.

Majzoub and associates assessed the seminal oxidation status (ORP) and SDF with respect to sperm morphology. Notably, sperm head abnormalities were far more common in infertile men than in fertile (54 percent vs. 48 percent). Furthermore, there are positive connections between ORP and SDF and sperm head defects. The motility and viability of sperm were negatively correlated with ROS and SDF (123).

Infertile men's seminal antioxidant capability and SDF appear to be positively correlated. The primary example utilized to exemplify the ROS-induced SDF theory is varicocele. Tahamtan et al. evaluated the telomeres of the sperm and leukocytes in infertile males with varicocele and also revealed the link between these two measures with oxidative stress and sperm functional tests (124). According to the Sakamoto et al. study, based on infertile males with OS and varicocele, a reduction in the levels of nitric oxide, 8-hydroxy-2′-deoxyguanosine, hexanoyl-lysine, and superoxide dismutase was observed after varicocelectomy (125).

A well-balanced diet is commonly known to include antioxidants, minerals, and vitamins. Therefore, it appears like a good strategy to administer antioxidants to lower ROS levels and enhance semen quality. Antioxidants as a therapeutic, however, are still being researched for their exact advantages, adverse effects, and dosage. Since antioxidants may be acquired online and without a prescription, they are regarded to be healthful supplements that are absolutely natural (126).

There is currently insufficient evidence to support the use of antioxidant supplements, according to a consensus statement of the European Society for Human Embryology. Recent evidence shows that vitamin C and E, zinc, folic acid, and selenium are the most widely used commercial supplements. The following table highlights the mechanism of action of various non-enzymatic antioxidants included in infertility supplements as well as their impact on semen parameters (127).

Numerous studies have attempted to determine whether antioxidant therapy would improve the characteristics of the sperm and, as a result, increase male fertility. Previous studies have shown that SDF has strong impacts on sperm integrity. Combining antioxidant therapy with L-carnitine, vitamin C, coenzyme Q10, vitamin E, zinc, folic acid, selenium, and vitamin B12 has enhanced sperm concentration rates and decreased SDF in men with low-grade varicocele. Furthermore, males who have asthenozoospermia, oligozoospermia, teratozoospermia, or enhanced baseline sperm concentrations may benefit from the use of carotenoids due to their better semen count and higher pregnancy rates. Supplementing with phytonutrients was found to raise sperm levels of SOD, catalase, and lipid peroxidation in the group of oligospermic males (128).

According to information available thus far, OS has a negative impact on semen integrity and, consequently, ART outcomes. Recent studies have shown that IVF and ICSI are both negatively impacted by ROS measurement in seminal plasma during conception (129). Vitamin E supplementation, especially in men with a history of unsuccessful IVF, may lead to better IVF outcomes than a placebo. Moreover, coenzyme Q10 supplementation boosts sperm concentration and motility, which results in successful ICSI (130).

Male infertility is occasionally brought on by OS, which results from an unbalance in antioxidants and ROS and leads to excessive SDF and aberrant semen parameters. The overproduction of ROS may be decreased as a result of antioxidant supplementation, which may also enhance the quality of the semen and increase the likelihood of clinical pregnancy, live births, and decreased miscarriage rates (131). However, it is strongly advised to conduct sizable, carefully crafted placebo-controlled studies to determine the precise effect of antioxidant supplements on male fertility.

Consuming antioxidants has been shown to prevent telomere shortening. Antioxidants are frequently utilized in male infertility treatment to balance out the seminal oxidative stress. According to a global survey's findings, 85.6% of doctors who treat male infertility recommend antioxidants as part of their regimen. Antioxidant consumption boosts sperm DNA integrity without any negative effects or issues, in addition to increasing semen parameters. In addition to these advantages, antioxidants can prevent somatic cells from losing their telomere length. Antioxidants alter proteins involved in CREM (cAMP responsive element modulator) signaling, mitochondrial activity, and protein oxidation at the subcellular level. Additionally, it has been claimed that they stimulate sperm's antioxidant defense mechanisms. Antioxidants are considered to help maintain telomere length throughout development by reducing the oxidative costs of growth, since telomeres are particularly vulnerable to damage brought on by OS. Antioxidants may be used therapeutically to lower ROS and enhance semen quality which may also benefit telomeres and infertility.

## Discussion

It has been established that telomere length is an indicator of cellular aging and reproductive maturity. Telomeres on leukocytes are distinct from those on sperm or oocytes and are not comparable to either. Age-related reductions in leukocyte telomere length occur, while unexpected gains in sperm telomere length occur (4). At the morula and blastocyst stages in the context of ART, fetal telomeres are hypothesized to reset their length. Telomeres, however, seem to be shorter than predicted in the beginning of development. The therapeutic approach to managing infertility may be enhanced by the assessment of TL in reproductive cells. Once it comes to the diagnosis and assessment of male and female infertility, we still don't have enough information on potential indicators.

Telomere homeostasis may exist when the ideal telomere length is maintained. As a result of telomere dysregulation, meiosis can be even more error-prone, leading to insufficient chromosomal segregation and a greater rate of aneuploidy. According to a report by Cariati and colleagues, pregnancy failures happen when male individuals have telomeres that are shorter than typical, supporting this theory (39).

Little research has specifically examined the relationship between STL and offspring telomere length. However, there is a clear paternal age impact on telomere length in a number of species, particularly rodents, birds, primates, and humans, according to which fathers who are older tend to have offspring with longer telomeres. Since STL has been associated with a number of sperm traits, it is obvious that STL cannot be utilized as the only determinant of the quality of sperm and embryos since it is difficult to determine the precise quantity of TL present in each male gamete that is capable of fertilizing an egg. The possibility for implantation and continuing pregnancy might be predicted, as well as the longevity of the children, by estimating the TL at the blastocyst stage.

Furthermore, because mitochondria are necessary for maintaining cellular health and vitality, stressful situations may affect their ability to maintain homeostasis. The primary process for preserving mitochondrial quantity and quality is mitophagy, which is supported by other specialized pathways. A few studies suggest a connection between several mitophagy processes that could work together to resist environmental stresses. Several physiological and pathological processes, including growth, differentiation, aging, cardiovascular disorders, neurodegenerative diseases, and cancer, are linked to mitochondrial dysfunction and poor mitophagy (132). The importance of regulating mitochondrial quality and quantity through mitophagy is brought home by the fact that impaired mitophagy contributes to aging and age—related diseases. Further research is necessary to determine the specific processes by which the many paths in the intricate network of mitophagy are connected.

Deficiency in chromatin condensation may be found in both sperm and somatic cells, and these deficiencies are caused by oxidative stress. By interfering with the chromosomes' non-random alignment in the sperm nucleus, the OS affects how the chromatin is packaged in sperm. An increase in telomere signals in the nucleus is caused by insufficient chromatin condensation as a result of the changed chromatin architecture. For the absence of chromosomal structure in the sperm nucleus, chromatin homeostasis disruption may be confirmed. Moreover, STL has been conclusively linked to the contamination of human sperm, correlating with the idea that shortened telomeres are more susceptible to attrition (133). However, abnormal semen traits, such as a low sperm count, poor motility, and increased DNA fragmentation, may be to blame for the short telomere length. For instance, one of the most prevalent reasons of male infertility is obesity, which is connected to STL shortening. Recent studies have suggested that lifestyle choices including drinking alcohol and coffee, exercising, and smoking contribute to the length of sperm telomeres. Atypical STL spermatozoa have not yet resulted in any successful pregnancies following IVF or ICSI, and the measurement of STL was not thought to be accurate in determining reproductive outcomes in ICSI cycles utilizing donor semen (53).

There is currently no consensus regarding the antioxidants that should be used or the dosage that should be used. However, it appears that the primary source of ROS for all different cell types is mitochondrial electron leakage (134). It may be helpful in this regard to enhance the development of antioxidants that particularly target the mitochondrial compartment, either by scavenging ROS within these organelles, which are known to promote ROS formation by the mitochondrial electron transport chain (135, 136). As always, the primary objective in the development of antioxidants is to lower the excessive production of damaging ROS metabolites while preserving the normal redox flow required for cellular activity. Inositol's beneficial effects on mitochondrial function, and therefore on sperm motility, may be attributable to its prokinetic activity, antioxidant and insulin-sensitizing capabilities, and hormonal regulating effects. For example, MI has a strong position in reproductive physiology since it positively affects the growth of oocytes, spermatozoa, and embryos. Despite significant advancements in our knowledge of the numerous aspects of telomere length as they relate to male reproduction, research has not yet shown a link between the spermatogenetic stage and epididymal sperm maturation and TL with advancing paternal age.

## Criteria

Inclusion and exclusion criteria are summarized in Table 2.

**Table 2:** The inclusion and exclusion standards used for this review.

Inclusion criteria	Exclusion criteria
Included studies must have compared certain treatments	Study was published in a language other than English
Included studies must have been published in the last 20 years	Study was published before 2,000 (exception basic literature)
**Database:** PubMed, NIH, Scopus, MEDLINE	Αrticles with questionable provenance
